# Efficacy of Digitalis in a Case of Pulmonary Hepatization

**Published:** 1845-06

**Authors:** 


*Efficacy of Digitalis in a case of Pulmonary Hepatization.*—Dr. Lauer relates in the *Medicinische Zeitung*, a case of pulmonary hepatization following pleuro-pneumony, in a Prussian soldier, successfully treated by a strong infusion of digitalis, with the addition of nitrate of potassa. Complete resolution of the hepatization was, he says, effected.

